# Associations between psychological factors and health-related quality of life and global quality of life in patients with ALS: a systematic review

**DOI:** 10.1186/s12955-016-0507-6

**Published:** 2016-07-20

**Authors:** Annerieke C. van Groenestijn, Esther T. Kruitwagen-van Reenen, Johanna M. A. Visser-Meily, Leonard H. van den Berg, Carin D. Schröder

**Affiliations:** Brain Center Rudolf Magnus and Center of Excellence for Rehabilitation Medicine, University Medical Center Utrecht and De Hoogstraat Rehabilitation, Utrecht, The Netherlands; Brain Center Rudolf Magnus, Department of Neurology, University Medical Center Utrecht, Utrecht, The Netherlands

**Keywords:** Amyotrophic Lateral Sclerosis, Quality of life, Health-related QoL, Global QoL, psychological factors, Moods, Beliefs, Personality

## Abstract

**Objective:**

To systematically identify and appraise evidence on associations between psychological factors (moods, beliefs, personality) and Health-related QoL (HRQoL) and/or global QoL in patients with Amyotrophic Lateral Sclerosis (ALS).

**Methods:**

A systematic review was conducted in several online databases (PsycINFO, EMBASE, PubMed and CINAHL) up to October 2015. Articles were included if they reported associations between psychological factors (moods, beliefs and personality) and HRQoL and/or global QoL in an ALS population. The search was limited to empirical studies, published in English, which provided quantitative data. The methodological quality of the included articles was assessed.

**Results:**

In total, 22 studies were included. Mood was investigated in 14 studies, beliefs in 11 studies and personality in one study. Fifteen different psychological factors were extracted and assessed using 24 different measures. Twelve different QoL measures were used in the selected studies, subdivided into seven different HRQoL measures and five different global QoL measures. Higher levels of anxiety and depression appeared to be related to a poorer HRQoL, whereas a higher level of religiosity seemed to be associated with better global QoL. No conclusive associations were found for confusion-bewilderment (mood), spirituality, mindfulness, coping styles, hopelessness, perception of burden, cognitive appraisal (beliefs), neuroticism, extraversion, openness, agreeableness and conscientiousness (personality), due to insufficient or inconsistent evidence. Religiosity and spirituality appeared to become more positively associated over time.

**Conclusions:**

Our results suggest that higher levels of anxiety and depression are related to a poorer HRQoL, whereas higher levels of religiosity appeared to be related to better global QoL. Associations might change during the disease course. This review supports the importance of psychological factors with regard to ALS care. Further research is needed to supplement the available evidence and to investigate how psychological factors can be modified to improve QoL.

**Review registration number:**

PROSPERO 2015:CRD42015027303

**Electronic supplementary material:**

The online version of this article (doi:10.1186/s12955-016-0507-6) contains supplementary material, which is available to authorized users.

## Background

Amyotrophic Lateral Sclerosis (ALS) is a fatal, progressive, neurodegenerative disorder affecting motor neurons in the spinal cord, brainstem and motor cortex. Patients suffer progressive wasting and weakness of limb, bulbar and respiratory muscles, leading to inability to speak and swallow, respiratory failure and complete paralysis [1, 2]. Currently, there is increasing awareness that ALS is also associated with non-motor findings, including behavioral and cognitive deficits [3, 4]. Patients eventually die due to respiratory failure within three to five years after symptom onset [1]. The incidence of ALS shows little variation in Western countries, ranging from 1.5 to 2.7 per 100,000 person-years, [5] with an estimated lifetime risk of 1 in 400 [6]. To date, no curative treatment is available. Therefore, optimal treatment is based on symptom management and optimizing Quality of Life (QoL).

There is, as yet, no agreed-upon definition of QoL. The World Health Organization (WHO) defines QoL as ‘a broad ranging concept affected in a complex way by the person’s physical health, psychological state, level of independence, social relationships and their relationship to salient features of their environment’ [7]. Burns et al. [8] have suggested that a distinction can be made between Health-related QoL (HRQoL) and global QoL in patients with ALS. Health-related QoL (HRQoL) is more narrowly defined than global QoL and seeks to address those aspects of self-perceived well-being that are related to or affected by the presence of disease or treatment [8]. Assessment of HRQoL typically includes physical, psychological and social domains. Each domain may include measures that assess the patient’s perception of symptoms, ability to function and disability [9]. Global QoL reflects overall QoL as judged by the patient and takes into account other, non-medical concepts, such as family, support system and friends [8]. Assessing global QoL generally provides a broader picture of the impact of disease on an individual’s life [9].

HRQoL declines during the course of the disease [10]. This is expected as HRQoL instruments are heavily weighted toward physical function, and thus inevitably decline over time as patients with ALS lose their abilities. In contrast, there is growing evidence that global QoL seems to remain at a stable level, even in patients with advanced ALS. Psychological processes like coping, reframing expectations and spiritual practice might contribute to a change in internal standards and values of QoL, ultimately resulting in unexpectedly high QoL, even in later disease stages [11, 12]. QoL in patients with ALS seems to be determined more by psychological, existential and support factors than by physical health [13–16], implying that a broad range of factors is involved in adjusting to illness. Psychological factors in patients with ALS may be modifiable targets for interventions to improve QoL.

The impact of psychological factors such as neuroticism, coping, cognitive appraisals and mood on QoL has already been demonstrated in other chronic diseases [17, 18] and in other progressive neurological illnesses, such as Huntington’s disease, Parkinson’s disease and multiple sclerosis [19]. Differences have, however, been reported between these progressive neurological illnesses and ALS concerning the contribution of psychological factors to QoL [19], suggesting that the rapidly progressive disabling process of ALS requires a different psychological adaptation process.

Over the last decade, interest has grown in the relationships between psychological factors and QoL in patients with ALS and to date, three narrative reviews on this subject have appeared [20–22]. The authors summarized associations between QoL and depression [20, 21], anxiety [20], spiritual and existential issues [20–22], sense of burden [22] and hope/hopelessness [20, 22] in patients with ALS, but as they did not quantify or appraise them, the relationships remain unclear.

The present study aims to collect and appraise the available evidence on the associations between psychological factors and HRQoL and/or global QoL. Understanding the relationships between QoL and psychological factors and the contribution of these factors to either HRQoL or global QoL might help health professionals to develop adequate interventions in order to optimize QoL in patients with ALS.

## Methods

This study was registered followed the PRISMA reporting guidelines (see online Additional file 1): 10.1371/journal.pmed.1000097

### Procedure

A search of online databases EMBASE, PsychINFO, PubMed and CINAHL was carried out up to October, 2015. No constraint was placed on the year of publication. The following MeSH headings and key words were used: ‘amyotrophic lateral sclerosis’ or ‘motor neuron disease’ in combination with ‘psychological factors’ (and synonyms including related terms, e.g. anxiety, depression, coping, religiosity and neuroticism) and ‘quality of life’ (and synonyms including related terms, e.g. well-being, value of life and perceived health). Additional file 2 provides an overview of the search strategy used in PubMed. Two authors (AvG, CS) independently checked the titles and abstracts on the selection criteria shown below, and compared their results. Concurrence between both researchers was calculated using Cohen’s kappa [23]. At each step of the process, disagreement regarding selection was discussed and settled with reference to the explicit inclusion criteria. If, after discussion, no agreement could be reached, another author (JV) was consulted for a final judgment. The same procedure was followed for final in—or exclusion after reading full text articles. The reference sections of retrieved articles were searched to identify further studies suitable for inclusion.

### Quality assessment

After the study selection, methodological quality was assessed independently by two researchers (AvG, EK) according to an 8-point checklist, resulting in a score that ranged from lowest quality (1) to highest quality (8) [17]. The level of agreement between the researchers’ ratings was established using the Intraclass Correlation Coefficient (ICC).

### Eligibility criteria and operationalization of concepts

The current review is restricted to empirical studies which provided quantitative data, thus excluding qualitative studies, reviews and case reports. Only studies of patients with ALS or providing separate data from patients with ALS were included, in which standardized measures were used to assess direct relationships between psychological factors (determinant) and a total QoL construct (outcome). Studies using a total score for HRQoL and global QoL, or a mental or physical component score of the HRQOL and / or a single-item score representing global QoL, were included. Thus studies describing associations between psychological factors and one subscale of a QoL measure were not taken into account. Furthermore, the review was limited to studies written in the English language that were published in peer-reviewed journals.

Psychological factors are part of the contextual factors (personal and environmental factors) defined by the International Classification of Disability, Functioning and Health (ICF) [24]. Psychological factors, such as coping styles, may play a role in disability at any level, but are not part of a health condition or health states [24]. In order to gain more insight into their association with HRQoL and/or global QoL, we have clustered the psychological factors into three main groups: mood, beliefs and personality. Mood is a generalized, internal state of feeling (e.g. anxiety, depression and anger) and is closely related to the concepts of affect and emotion. Beliefs refer to people’s perceptions of reality including perceptions of health or illness and one’s ability to cope with illness (e.g. attitudes, appraisals, religiosity and coping strategies). Personality can be defined as a dynamic and organized set of characteristics which a person possesses and which uniquely influence his or her beliefs, motivations and behaviour in various situations [25].

### Data extraction and analysis

We collected information on study characteristics: author, country, sample size and study design, and patient characteristics: age at inclusion, the time of assessment since ALS onset, the functional status of patients (using the Amyotrophic Lateral Sclerosis Functional Rating Scale (ALSFRS), the diagnostic criteria and the type of ALS onset (spinal vs. bulbar). Furthermore, measures of global QoL and HRQoL and of psychological factors, as well as associations between psychological factors and QoL (Health-related and global) were extracted.

Bivariate and multivariate associations were described separately in terms of correlation coefficients (r), standardized β-coefficients (β) and the explained variance of the psychological factors (*R*^2^). The strength of correlation was described as follows: “weak” correlation = 0 < ∣r∣ < 0.3; “moderate” correlation 0.3 < ∣r∣ < 0.5; “strong” correlation ∣r∣ > 0.5 (Cohen, [23]). We classified the methodological quality of the studies to be “high” if they were above 5.5, “adequate” between 3.5 and 5.5, and “poor” below 3.5.

Psychological factors were considered “consistent related” if (1) the majority (>50 %) of all studies reported statistically significant bivariate and/or multivariate associations; (2) the majority of the bivariate associations were moderate or strong; and (3) the methodological quality of these studies was adequate or high.

## Results

### Description of studies included

**Fig. 1:**
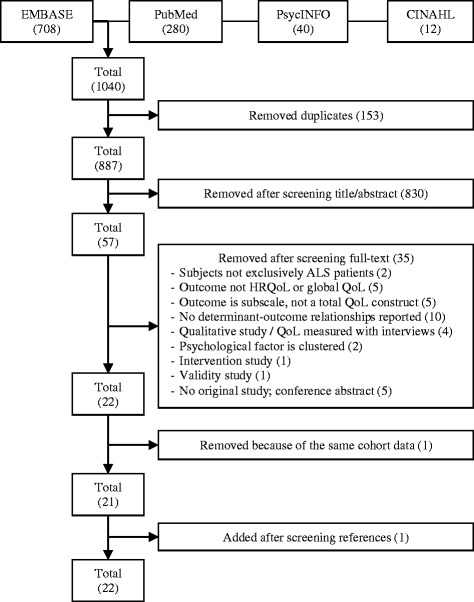
Search flowchart

The search strategy produced a total of 1040 articles (Fig. 1). After removing 153 duplicates, a further 830 articles were removed after screening title and abstract. Agreement on selection of titles and abstracts between the two raters was high (Cohen’s kappa 0.82). A total of 57 articles remained for full-text screening; 22 articles met all inclusion criteria. The screening of reference lists produced one additional article [10]. In two studies [16, 26], the same cohort data was used; we included the study by Bremer because of a higher quality assessment.

The characteristics of the 22 included studies are presented in Table 1. Studies were published between 1999 and 2015; most were cross-sectional (*n =* 16); six used longitudinal data [16, 27–31]. The median sample size was *n =* 49 (range 26–197). Studies concerned patients with a mean time of ALS onset between 11.7 months and 5.7 years; the disease severity ranged from 17.4 (severely impaired) to 35.1 points (moderately impaired). The mean age at inclusion varied between 55.3 and 64.0 years; a minority of the patients (7-33 %) had a bulbar onset of ALS, and there was a slight male predominance (M:F ratio ~ 1.5:1). These findings were consistent with those of the general ALS population (mean age 58–63 years; bulbar onset of 30 % and M:F ratio ~ 1.2–1.5:1) [32, 33].

**Table 1:** Patient and study characteristics

	Study characteristics			Patient characteristics				
Author (date)	Country of research	Sample size n (male n)	*Design*	*Age in years*	Time since onset (O)/ Time since diagnosis (D)	ALSFRS	Diagnostic criteria	Onset bulbar (%)
Bremer (2004)^T0^ [16]	U.S.A	49 (29)	Longitudinal	57.8 (13.0)	34.9 (13.2) mo^O^	27.9 (6.3)	El Escorial	n.m
Chio (2004) [13]	Italy	80 (49)	Cross sectional	59.8 (12.6; 26–81)	2.1 (1.7; 1–7.8) yr^O^	26.6 (9.5; 3–38)	El Escorial	n.m.
Clarke (2001) [15]	Ireland	26 (18)	Cross sectional	63^M^ (34–86)	31.5 (4–156)^M^ mo^D^	22.5^M^ (11–36)	El Escorial	n.m
Dal Bello-Haas (2000) [47]	U.S.A	60 (38)	Cross sectional	56.2 (12.2)	n.m	n.m	El Escorial	n.m.
Ganzini (1999) [45]	U.S.A.	100 (61)	Cross sectional	54^M^ (51.6–56.8)	2.8 (2.0–3.6)^M^ yr^D^	n.m	n.m	n.m
Gibbons (2013) [37]	U.K.	147 (90)	Cross sectional	61 (11; 35–81)	n.m	22.3 (9.5; 4–48)	*‘confirmed diagnosis’*	n.m
Goldstein (2002) [38]	U.K	31 (19)	Cross sectional	64.0 (11.9)	15.9 (5.2) mo^D^	n.m	El Escorial	n.m
Ilse (2015) [10]	Germany	49 (25)	Cross sectional	63.8 (10.0)	35.1 (36.3) mo^O^	32.6 (9.2) ^R^	El Escorial	33 %
Krampe (2008)^T0^ [27]	Germany	31 (19)	Longitudinal	60.3 (10.4; 32.9–79.7)	96.3 (70.5; 22.4–330.4) wk^O^	27.0 (6.6; 12–38)	El Escorial	19 %
Lule (2008)^T0^ [28]	Germany	39 (19)	Longitudinal	n.m	43.9 (37.5; 0–170) mo^D^	19.9 (21.1; 0–39)	El Escorial	n.m
Matuz (2010) [36]	Germany	27 (15)	Cross sectional	55.3 (11.1; 35–73)	36 (4–129) mo^D^	17.4 (9.8; 0–36)	*‘by a neurologist’*	7 %
Matuz ^T0^ (2015) [30]	Germany	27 (15)	Longitudinal	55.3 (11.1; 35–73)	43.2 (30.5; 4–129) mo^D^	17.4 (9.8; 0–36)	El Escorial	7 %
McCabe (2009) [19]	Spain	120 (72)	Cross sectional	63.2 (12.4)	5.7 (5.8)^O^ yr	n.m	n.m	n.m
Montel (2012) [46]	France	49 (26)	Cross sectional	63 (12)	45 (28) mo^O^	28.2 (9.0)^R^	El Escorial	22 %
Pagnini ^T0^ (2015) [29]	Italy USA	197 (115)	Longitudinal	^a^	^a^	30.6(9.9)^SA^	‘self-declared’	n.m
Peric (2010) [40]	Serbia	74 (45)	Cross sectional	57 (11)	29 (27) mo^O^	34 (8)^R^	El Escorial	n.m.
Pizzimenti (2013) [43]	Italy	36 (22)	Cross sectional	63.7 (10.9)	22 (14) mo^O^	35.1 (8.7)	El Escorial	22 %
Robbins (2001)^T0^ [31]	U.S.A	60 (32)	Longitudinal	58.5 (13.5; 27–83)	n.m	28.1 (6.3; 12–39)	El Escorial	n.m
Simmons (2000) [14]	U.S.A	96 (52)	Cross sectional	57.8 (23–80)	31.8 (2 mo-10 year) mo^O^	26.6 (9–39)	*‘met the criteria’*	n.m
Tramonti (2012) [42]	Italy	40 (30)	Cross sectional	59.1 (10.9; 34–84)	n.m	20.8 (8.3; 7–36)	n.m	n.m
Vignola (2008)^TD^ [41]	Italy	29 (20)	Cross sectional	63.6 (7.8; 44–78)	11.7 (23.7; 2–43) mo^O^	33.1 (4.8; 22–39)	El Escorial	n.m
Winter (2010) [44]	Germany	37 (21)	Cross sectional	59.6 (11.0)	2.3 (1.9) yr^O^	n.m	El Escorial	n.m

*Note.* Numbers are presented as means (SD; range), unless stated otherwise

Abbreviations: *n.m* not mentioned, *M* median instead of mean, *yr* year, *mo* months, *wk* weeks, *ALSFRS* Amyotrophic Lateral Sclerosis Functional Rating Scale, *R* ALSFRS-Revised, *SA* self-administered ALSFRS, *T0* data from baseline measurement, *TD* data from diagnostic phase, *O* onset, *D* diagnosis

^a^ time since diagnosis and age were reported in *categories*

Across the studies, fifteen different psychological factors were assessed using 24 different measures. The various instruments for assessing psychological factors are described in Table 2. Mood was investigated in 14 studies, beliefs in 11 and personality in one study. Two ALS-specific questionnaires, the ALS Depression Inventory (ADI-12) [34] and the Motor Neuron Disease Coping Scale (MNDCS) [35] were applied in one [28] and three [30, 36, 37] studies, respectively. The modified versions of the Hospital Anxiety and Depression Scale (HADS), which were intended not to rely on measuring the physical components of depression, were used in two studies [37, 38] (Table 2).

**Table 2:** Psychological factor measurements

Psychological factors	Measurement and references	Number of items	Description	Scoring system	Generic/ALS-specific measure	References in this review
Anxiety	Hamilton rating scale for anxiety (HAM-A) Hamilton (1959) [63]	14	To assess the severity of symptoms of anxiety. Each of the items contains a number of symptoms, and each group of symptoms is rated on a scale.	5-point scale. Total scores for anxiety range from 0 to 56. Score interpretation: <17: mild severity 18–24: mild to moderate severity 25–30: moderate to severe	Generic	[40]
	Hospital Anxiety and Depression Scale subscale anxiety (HADS-a) Zigmond (1983) [54]	7	To assess psychological distress in medically ill patients. The instrument concentrates on the psychic rather than the somatic symptoms of mood disorder in order to provide an assessment of mood independent of levels of physical disability in patients with medical illnesses.	4-point scale. Total scores for anxiety range from 0 to 21 Score interpretation: ≤7: non-cases 8–10: possible clinical levels of distress 11–21: clinical levels of distress	Generic	[15, 38]
	Hospital Anxiety and Depression Scale subscale anxiety (HADS-a)—*modified version 1* Gibbons (2011) [56]	6	To assess psychological distress in medically ill patients. The original HADS was modified with removal of question 11 of the original HADS “I feel restless as if I have to be on the move”.	4-point scale. Total scores for anxiety range from 0 to 18. Score interpretation: Scores of 9–18: case level anxiety	ALS	[37]
	State and Trait Anxiety Inventory (STAI) Spielberger (1968) [64]	40	To assess trait and state anxiety. STAIs: 20 items assess trait anxiety. STAIs is defined as an unpleasant emotional arousal in face of threatening demands or dangers; STAIt: 20 items assess state anxiety. STAIt reflects the existence of stable individual differences in the tendency to respond with state anxiety in the anticipation of treating situations. These two parts differ in the item wording, in the response format (intensity versus frequency), and in the instructions given for responses.	4-point scale. Total scores for anxiety (STAIs and STAIt) range from 20 to 80. Score interpretation: 20–39: low anxiety 40–59: medium anxiety 60–80: high anxiety	Generic	[41]
Depression	Beck Depression Inventory (BDI) Beck (1961) [53]	21	To assess severity of depressive symptoms.	4-point scale. Total scores for depression range from 0 to 63. Score interpretation: 0–9: no depressive symptoms 10–18: mild to moderate depressive symptoms 19–29: moderate to severe depression 30–63: severe depression	Generic	[10, 27, 44]
	Hamilton rating scale for depression (HAM-D) Hamilton (1960) [63]	21	To assess patient’s level of depression. The first 17 of the 21 items contribute to the total score and items 18–21 give additional information, not part of the scale, such as paranoia and diurnal variation	8 items 5-point scale; 9 items 3-point scale. Total scores for depression range from 0 to 50. Score interpretation: 0–7 = normal 8–13 = mild depression 14–18 = moderate depression 19–22 = severe depression 23–50 = very severe depression	Generic	[40]
	Hospital Anxiety and Depression Scale subscale depression (HADS-d) Zigmond (1983) [65]	7	To assess psychological distress in medically ill patients. The instrument concentrates on the psychic rather than the somatic symptoms of mood disorder in order to provide an assessment of mood independent of levels of physical disability in patients with medical illnesses.	4-point scale. Total scores for depression range from 0 to 21. Score interpretation: ≤7: non-cases 8–10: possible clinical levels of distress 11–21: clinical levels of distress	Generic	[15]
	Hospital Anxiety and Depression Scale subscale depression (HADS-d) - *modified version 2* Abrahams (1997) [66]	6	To assess psychological distress in medically ill patients. The original HADS was modified with removal of question 8: “I feel slowed down”, as it was felt likely that this would falsely exaggerate the measure of depression due to the physical symptoms of ALS.	4-point scale. Total scores for depression range from 0 to 18. Score interpretation: ≤7: non-cases 8–10: possible clinical levels of distress 11–21: clinical levels of distress	ALS	[38]
	Hospital Anxiety and Depression Scale subscale depression (HADS-d) - *modified version 1* Gibbons (2011) [56]	6	To assess psychological distress in medically ill patients. The original HADS was modified with removal of question 8: “I feel slowed down”.	4-point scale. Total scores for depression range from 0 to 16 (Two items in the depression subscale were recorded 0-1-1–2). Score interpretation: Scores of 8–16: case level depression.	ALS	[37]
	Depressive disorders: DSM-IV. American Psychiatric Association (2000) [67]	9	To assess a major depressive disorder.	Score interpretation: 5 out of 9 symptoms have to be present and represent a change from previous functioning; at least one of the symptoms is either (1) depressed mood or (2) loss of interest or pleasure.	Generic	[45]
	ALS-Depression-Inventory (ADI-12) Kubler (2005) [34]	12	To assess depressive symptoms, specifically developed for ALS patients and addresses depressive symptoms excluding increasing physical impairments commensurate with ALS.	4-point scale. Total scores for depression range from 12 to 48. Score interpretation: <22: absence of depression 22–28: mild depression >28: clinically relevant depression	ALS	[28]
	Zung Depression Scale (ZDS); Zung (1965) [68] also called Zung Self-Rating Depression Scale (SDS) (1965)	20	To assess depression	4-point scale. Total scores for depression range from 20 to 80 Score interpretation: 50–59: mild depression 60–69: moderate depression 70–80: severe depression	Generic	[13, 41–43]
Mood	Profile of Mood State short-form (POMS-SF) McNair (1992) [69]	37	To assess six states of mood: tension-anxiety, depression-dejection, anger-hostility, vigour-activity, fatigue-inertia, and confusion-bewilderment.	Abbreviated 37-item version of the original scale using the 5-point Likert Scale; 0 (not at all) to 4 (extremely). Total mood disturbance (TMD): sum of the subscales. Score interpretation: Higher scores reflect higher presence of the mood state.	Generic	[19]
Religiosity	Idler Index of Religiosity (IRR) Idler (1987) [70]	4	To assess the level of religiosity. It addresses both public and private aspects of religiosity: Public religiosity (IIR-Pu) (2 items): frequency of church attendance and number of church members known personally Private religiosity (IIR-Pr) (2 items) how religious they perceived themselves to be and the amount of strength and comfort obtained from religious practices.	Public religiousness: 1-item, 6-point scale; 1-item, 4-point scale Private religiousness: 1-item, 4- point scale; 1-item, 3-point scale The religiosity scores are summed to produce public, private, and total religiosity; scores range from 2–10, 2–7 and 4–17, respectively. Score interpretation: Higher scores indicate higher level of religiosity.	Generic	[13, 14, 16, 31]
Spirituality	Spiritual Well-being Scale (SWBS) (1) Reed (1987) [71]	10	To assess the level of spiritual well-being.	6-point scale. Total scores of spiritual well-being range from 6 to 36. Score interpretation: Higher scores indicate higher spiritual well-being.	Generic	[16]
	Spiritual Well-being Scale (SWBS) (2) Ellison (1983) [72]	20	The scale consists of 10 religious well-being items (RWB) and 10 existential well-being items (EWB); spiritual well-being.	6-point scale. Total scores (RWB + EWB) of spiritual well-being range from 20 to 120. Score interpretation: Higher scores indicate higher spiritual well-being.	Generic	[47]
Mindfulness	Langer mindfulness scale (LMS) Pirson (2012) [73]	14	Three domains associated with mindful thinking: novelty seeking, engagement and novelty producing.	Total scores range from 14–98, Score interpretation: Higher scores reflect higher mindfulness	Generic	[29]
Hopelessness	Becks Hopelessness Scale (BHS) Beck (1974) [74]	20	To assess three major aspects of hopelessness: feelings about the future, loss of motivation, and expectations.	2-point scale. Total scores of hopelessness range from 0 to 20. 8–13: moderate hopelessness >14: severe hopelessness Score interpretation: Higher scores reflect higher levels of hopelessness.	Generic	[45]
Perception of burden to others	Zarit Burden Inventory (ZBI) – *revised* Zarit (1980) [75]	3	Three items of the original ZBI were revised to measure patient beliefs that their medical condition stressed, burdened, or caused financial hardship to their family.	5-point scale. Total score of perception of burden to others (1 item) range from 0 to 4. Score interpretation: Higher score indicates higher perception of burden to their family.	ALS	[45]
Cognitive appraisal	Appraisal scale Smith (1993) [76]	4	To assess patients’ primary (motivational relevance, motivational congruence) and secondary appraisal (problem-focused and emotion-focused coping potential)	9-point scale. Scores per item range from 1 to 9. Total scores are not mentioned. Score interpretation: The larger the difference between the two items of primary appraisal (motivational relevance and motivational congruence), the more patients feel threatened by the disease.	Generic	[30, 36]
Coping	Motor Neuron Disease Coping Scale (MNDCS) – *adapted version 1* Lee (2001) [35]	18	To assess extent to which patients relied on the coping strategies. 18 questions of the original 22-item scale were assigned to 6 subscales.	6-point scale. Total score for each type of coping was obtained by generating the mean score of the grouped scales. Ranges of total scores are not mentioned. Score interpretation: Higher score reflects greater use of the coping strategy.	ALS	[30, 36]
	Motor Neuron Disease Coping Scale (MNDCS/Cope-MND)—*adapted version 2* Lee (2001) [35]	9	To assess extent to which patients relied on the coping strategies. The original MNDCS was reduced to a 9-item scale.	6-point scale. Ranges of total scores are not mentioned. Score interpretation: Higher scores reflect greater use of the coping strategy.	ALS	[37]
	The Brief COPE Carver (1997) [77]	28	Measures 14 dimensions of coping: distraction; active coping; denial; emotional support; instrumental support; disengagement; venting; positive reframing; planning; acceptance; humour; religion; self-blame; substance use. Each dimension consists of 2 items.	4-point scale. No overall score. Score range per dimension ranges from 2 to 8, per item from 1 to 4. Score interpretation: Higher score reflects greater use of the coping strategy.	Generic	[46]
Personality traits	NEO Five Factor Inventory (NEO-FFI) Costa (1992) [78]	60	To assess the five dimensions of personality, postulated by the five-factor model of personality: neuroticism, extraversion, openness, agreeableness, and conscientiousness.	5-point scale. Each of the five-factor subscales consists of 12 items, resulting in mean factor scores ranging from 0 to 4. Score interpretation: Higher score reflects a type of personality.	Generic	[27]

A total of 12 different QoL measures were used in the selected studies, including five different Global QoL measures and seven different Health-related QoL measures (Table 3 and Table 4, resp.). One ALS-specific HRQoL questionnaire, the Sickness Impact Profile ALS (SIP/ALS-19) [39], was used in two studies [14, 31].

**Table 3:** Global Quality of Life measurements

Global QoL Measurement and reference	Number of items	Description	References in this review
The Schedule for the Evaluation of Quality of Life (SEIQoL) McGee (1991) [79] O’Boyle (1992) [80]	46	SEIQoL assesses overall subjective QoL as judged by the patient in healthy or ill individuals. It is derived from a decision analysis technique known as judgement analysis, administered through a semi-structured interview. Patients rate their satisfaction with areas of their life by assessing three aspects of QoL. The patients have to 1) nominate the life areas (cues) which are important to their QoL; 2) rate their current level of functioning in each of these salient areas; and 3) rate the relative importance of each of their chosen cues. SEIQoL index score: the SEIQoL scores are entirely person-specific, for the purpose of group analyses an overall global or index QoL score (also referred to as a total QoL score) is calculated. The resulting SEIQoL index ranges from 0 (worst possible QoL) to 100 (best possible QoL).	[15, 42]
The Schedule for the evaluation of Quality of Life-Direct Weighting (SEIQoL-DW) Hickey (1996) [81]	15	SEIQoL-DW is a shorter, direct-weight (DW) version of the SEIQoL, employs an alternative method of deriving cue weights using a colored disk. SEIQoL-DW index score: The SEIQoL-DW scores are entirely person-specific; for the purpose of group analyses an overall global or index QoL score (also referred to as a total QoL score) is calculated. The resulting SEIQoL index ranges from 0 (worst possible QoL) to 100 (best possible QoL).	[13, 28, 30, 38]
The McGill Quality of Life Questionnaire (MQOL) Cohen SR (1995/1996) [82, 83]	17	MQOL assesses overall subjective QoL as judged by the patient. Subjects evaluate their lives over the past 2 days on five subscales using a 10-point semantic-differential format. Originally designed for cancer and HIV patients. It is not heavily weighted toward physical function and it includes an existential element. MQOL includes five domains, two of which are health-related: Physical Symptoms (MQOL-Ph) (3 items) and Physical Well-being (MQOL-PW) (1 item); and three are non-health related: Psychological symptoms (MQOL-Ps) (4 item); Existential Well-being (MQOL-EW) (6 items) and Social Support (MQOL-Su) (2 items). Scores on the subscales range from 0 (worst) to 10 (best). The MQOL total score is the mean of the 5 subscales, score ranges from 0 (worst QoL) to 10 (best QoL). MQOL-SIS: besides the subscales there is also a Single-Item Score (SIS): the patient is asked to indicate his/her self-perceived overall QoL in the past two days in a single-item scale (SIS) measuring overall subjective QoL, rated from 0 (very bad) to 10 (excellent).	[13, 14, 16, 27, 29, 41]
QoL-single-item question Self-developed by Ganzini (1999) [45]	1	A single-item question to assess patients self-perceived overall QoL. End-points labelled 1 = “my quality of life is as good as it can be” and 6 = “my quality of life is very bad, horrible”.	[45]
QoL-single-item question Self-developed by Krampe (2008) [27]	1	A single-item question to assess patients self-perceived overall QoL. “Over the past seven days, the quality of my life has been”: very poor (0) – excellent (10).	[27]

**Table 4:** Health-related Quality of Life measurements

Health-related QoL Measurement and reference	Number of items	Description	References in this review
The 36-items Short Form of the Medical Outcomes Study questionnaire (SF-36) Ware (1993) [52]	36	SF-36 is a standardised, generic health-related quality of life measure. It consists of 36 items covering 8 dimensions. Each dimension is transformed into a 0–100 scale on the assumption that each question carries equal weight. High scores indicate good QoL. Four of these dimensions (limitations in physical functioning (PF); role limitations due to physical health problems (RP), bodily pain (BP), and general health perceptions (GH)) are summarized in the Physical Component Score (PCS), and four others (vitality (VT); social functioning (SF), role limitations due to emotional problems (ER), general mental health (MH)), in the Mental Component Score (MCS).	[40, 44, 46]
Sickness Impact Profile (SIP) Bergner (1981) [84]	136	SIP measures physical, mental and social aspects of health-related functioning; it contains statements regarding behaviour “sickness impact” and the individual is asked to respond by checking items that describe their health status. SIP contains 136 items in 12 categories and two dimensions (physical and psychosocial). Overall, category and dimension scores may be calculated from 0—100 (best).	[47]
Sickness Impact Profile (SIP/ALS-19) McQuire (1997) [39]	19	SIP/ALS-19 assess health-related QoL. It is a questionnaire consisting of 19 items from the full SIP (Sickness Impact Profile) believed to have the greatest impact on QoL, based on opinions of ALS clinical specialists. Extracted from the full SIP total score range from 0—100 (best).	[14, 31]
EuroQoL-5D Brazier (1993) [85]	5	EuroQoL-5D assess health-related QoL. It consists of five questions that relate to five dimensions of health: mobility, self-care. usual activities, pain/discomfort, anxiety/depression. Each dimension is divided into three levels of severity (1, no problem; 3 severe problem). The EQ-5D-index score can be calculated.	[10, 44]
EQ VAS Konig (2005) [86]	1	EQ VAS assess health-related QoL. It is a visual analogue scale (VAS thermometer type) to rate patients current HRQoL ranging from 0 (worse imaginable health state) to 100 (best imaginable health state).	[44]
World Health Organization Quality of Life brief questionnaire (WHOQoL-BREF) Skevington (2004) [87]	26	WHOQoL-BREF assesses quality of life within the context of an individual’s culture, value systems, personal goals, standards and concerns. Generic instrument, measures QoL of life across 4 domains: physical health (7 items), psychological health (6 items), social relationships (3 items) and environment (8 items). Domain scores can be transformed to total scores from 0 (worse imaginable health state) to 100 (best imaginable health state). Two other items measure overall QoL and general health. Items are rated on a 5-point scale (low score of 1 to high score of 5) to determine a raw item score. Subsequently, the mean score for each domain is calculated, resulting in a mean score per domain that is between 4 and 20. Finally, this mean domain score is then multiplied by 4 in order to transform the domain score into a scaled score, with a higher score indicating a higher QoL.	[19, 37]
Quality of Life Index (QL-Index) Spitzer 1981 [88]	5	The Spitzer Qol Index (SQLI/ QLI/ QL-Index) assesses health-related QoL in palliative care populations. It covers five dimensions of quality of life: activity, daily living, health, support of family and friends, and outlook on life. Each dimension is rated on a three-point Likert scale (0 to 2), with the range of scores from 0 to 10. Lower scores reflect a higher QoL.	[43]

The average methodological quality score of the studies was 5.3 and ranged from 3 to 8 out of a maximum 8 points (Table 5). Seven studies (32 %) achieved a “high quality” score (≥6/8). Inter-rater agreement on quality of the individual studies was high (ICC = 0.90).

**Table 5:** Methodological quality assessment

Reference	Year	Internal validity	Control of drop out	External validity	Statistical validity	Proportion Sample size vs determinants	Multi-collinearity	Confounding bias	Reporting	Total (max 8 points)
Bremer [16]	2004	1	1	1	1	0	0	0	1	5.0
Chio [13]	2004	1	0.5	1	1	0	0	1	1	5.5
Clarke [15]	2001	1	1	1	1	0	0	0	1	5.0
Dal-Bello-Haas [47]	2000	1	0	0.5	1	0	0	0	1	3.5
Ganzini [45]	1999	0	1	0.5	1	1	0	1	1	5.5
Gibbons [37]	2013	1	0	1	1	1	1	1	1	7.0
Goldstein [38]	2002	1	0	1	1	0	0	0	0	3.0
Ilse [10]	2015	1	0	1	1	0	0	0	1	4.0
Krampe [27]	2008	0	0	1	1	0	0	1	1	4.0
Lule [28]	2008	1	0	0.5	1	0	0	1	1	4.5
Matuz [36]	2010	1	0	1	1	0	1	1	1	6.0
Matuz [30]	2015	1	1	1	1	0	0	1	1	6.0
McCabe [19]	2009	1	0	0.5	1	1	0	1	1	5.5
Montel [46]	2012	1	0	1	1	0	0	0	1	4.0
Pagnini [29]	2015	1	0.5	0.5	1	1	0	1	1	6.5
Peric [40]	2010	1	0.5	1	1	0	1	1	1	6.5
Pizzimenti [43]	2013	1	1	1	1	1	1	1	1	8.0
Robbins [31]	2001	1	0.5	0.5	1	1	0	1	1	6.0
Simmons [14]	2000	1	0.5	1	1	0	0	0	1	4.5
Tramtoni [42]	2012	1	0	0.5	1	0	0	1	1	4.5
Vignola [41]	2008	1	0.5	1	1	0	0	1	1	5.5
Winter [44]	2010	1	0.5	0.5	1	0	0	1	1	5.0

*Note.* 1 = internal validity: use of validated and reliable measures, 2 = control of patient drop-out: including nonresponse analysis and describing executive patients, 3 = external validity: specifying in/exclusion criteria and demographic and disease characteristics (diagnosis, age, gender, site of ALS onset, time since diagnosis, severity), 4 = statistical validity: testing for statistical significance, 5 = adequate sample size in relation to the number of determinants (univariate ratio 20:1 and multivariate ratio 10:1), 6 = control for multicollinearity, 7 = control for potential confounding variables, 8 = clear description of main finding [17]

### Psychological factors associated with QoL in ALS

An overview of the bivariate and multivariate associations between psychological factors and QoL is presented in Table 6. Due to the heterogeneity of instruments used in assessing both psychological factors (*n =* 24) and QoL (*n =* 12), a meta-analysis was not possible.

**Table 6:** Results of the bivariate and multivariate associations between psychological factors and QoL in patients with ALS

	Psychological Factor	Measure Psychological factor		Measure QoL	Time-points of assessment/F.U./trajectory	Bivariate Association *r*		Multivariate Association β/R^2^	Ref.	Quality Score (max. 8)
MOOD	**Anxiety**									
	Anxiety	HAM-A	HRQoL	SF-36 total		ns/nr			[40]	6.5
		HAM-A		SF-36 PCS		ns/nr				
		HAM-A		SF-36 MCS		ns/nr				
		HADS-a^1^		WHOQoL-BREF total		−0.53**			[37]	7.0
	Anxiety - Tension	POMS		WHOQoL-BREF total		nr		β = −0.47*	[19]	5.5
	Anxiety	HADS-a	Global QoL	SEIQoL index score		ns/nr			[15]	5.0
		HADS-a		SEIQoL-DW index score		ns/nr			[38]	3.0
	State anxiety	STAIs^O^		MQOL total		nr		s**/nr	[41]	5.5
		STAIs		MQOL total	<1 mo after D.	nr		s**/nr		
		STAIs		MQOL total	>1 mo after D.	nr		s*/nr		
	Trait anxiety	STAIt		MQOL total	<1 mo after D.	nr		s**/nr		
		STAIt		MQOL total	>1 mo after D.	nr		s* /nr		
	Depression									
	Depression—Dejection	POMS	HRQoL	WHOQoL-BREF total		nr		β = −0.24*	[19]	5.5
	Depression	HADS-d^1^		WHOQoL-BREF total		−0.60**		β = −0.41*	[37]	7.0
		HAM-D		SF-36 total		ns/nr			[40]	6.5
		HAM-D		SF-36 PCS		ns/nr				
		HAM-D		SF-36 MCS		ns/nr				
		ZDS		SF-36 total		−0.617**			[42]	4.5
		ZDS		QL-Index		s* /nr		β = −0.08*	[43]	8.0
		BDI		EQ-5D index score		−0.430**			[10]	4.0
		BDI^A^		SF-36 MCS		nr		β = −0.391**	[44]	5.0
		BDI^A^		SF-36 PCS		ns/nr				
		BDI^A^		EQ-5D index score		nr		β = −0.272		
		BDI^A^		EQ VAS		nr		β = −0.381*		
		BDI		HRCS	1 mo F.U.	nr		s / nr	[27]	4.0
		BDI		HRCS	12 mo F.U.	nr		s**/nr		
	Depression x time	BDI		HRCS	over 12 mo	nr		ns		
	Depression	ADI-12	Global QoL	SEIQoL index score		−0.36*			[28]	4.5
		DSM-IV		Single-item-question^3^		ns/nr		ns / nr	[45]	5.5
		ZDS		SEIQoL-DW index score		nr		s*/nr	[13]	5.5
		ZDS		MQOL Single Item Score		nr		s*/nr		
		ZDS		SEIQoL index score		−0.205			[42]	4.5
		ZDS^o^		MQOL total		nr		s**/nr	[41]	5.5
		HADS-d		SEIQoL index score		ns/nr			[15]	5.0
		HADS-d^2^		SEIQoL-DW index score		ns/nr			[38]	3.0
		BDI		Single-item-question^4^	1 mo F.U.	nr		s*/nr	[27]	4.0
		BDI		Single-item-question^4^	12 mo F.U.	nr		s*/nr		
	Depression x time	BDI		Single item question^4^	*over* 12 mo	nr		ns		
	Confusion - Bewilderment	POMS	Global QoL	WHOQoL-BREF total		nr		β = 0.33*	[19]	5.5
BELIEFS	Religiosity									
	Religiosity	IIR-tot	HRQoL	SIP/ALS-19 total		0.169			[14]	4.5
		IIR-tot		SIP/ALS-19 total	3 mo F.U.	nr		ns / nr	[31]	6.0
		IIR-tot		SIP/ALS-19 total	6 mo F.U.	nr		s***/nr		
	Religion - coping	Brief COPE		SF-36 PCS/SF-36 MCS		−0.26^P^	−0.01^M^		[46]	4.0
	Religiosity	IIR-tot	Global QoL	MQOL total		0.15			[16]	5.0
		IIR-tot		MQOL total	3–4 mo F.U.	0.28				
		IIR-tot		MQOL total	6–8 mo F.U.	0.37**				
		IIR-tot		MQOL total	9–12 mo F.U.	0.33*				
		IIR-tot		MQOL total	12–16 mo F.U.	0.46**				
		IIR-tot		MQOL total	3 mo F.U.	nr		ns / nr	[31]	6.0
		IIR-tot		MQOL total	6 mo F.U.	nr		ns / nr		
		IIR-tot		MQOL total		0.221			[14]	4.5
		IIR-tot		MQOL Single Item Score		0.331**				
	Religiosity Private	IIR-Pr		MQOL total		0.13		β = 0.05; R^2^ = 0%	[16]	5.0
		IIR-Pr		MQOL total	3–4 mo F.U.	0.42**		β = 0.31**		
		IIR-Pr		MQOL total	6–8 mo F.U.	0.49**		β = 0.35**		
		IIR-Pr		MQOL total	9–12 mo F.U.	0.34*		β = 0.21		
		IIR-Pr		MQOL total	12–16 mo F.U.	0.50**		β = 0.41***; R^2^ = 16 %***		
		IIR-Pr		SEIQoL-DW index score		s*/nr			[13]	5.5
	Spirituality								[16]	5.0
	Existential well-being	SWBS^6^ - EWB	HRQoL	SIP total		ns/nr			[47]	3.5
	Religious well-being	SWBS^6^ - RWB		SIP total		−0.996**				
	Spiritual well-being	SWBS^5^ total	Global QoL	MQOL total		0.08			[16]	5.0
		SWBS^5^ total		MQOL total	3–4 mo F.U.	0.08				
		SWBS^5^ total		MQOL total	6–8 mo F.U.	0.17				
		SWBS^5^ total		MQOL total	9–12 mo F.U.	−0.12				
		SWBS^5^ total		MQOL total	12–16 mo F.U.	0.54**				
	Mindfulness									
	Mindfulness	LMS	Global QoL	MQOL Single Item Score		nr		β = 0.06***	[29]	6.5
	Mindfulness x time	LMS		MQOL Single Item Score	*over* 4 mo	nr		β = 0.009		
	Coping									
	Positive coping strategies	MNDCS^1^	HRQoL	WHOQoL-BREF total		0.46**		β = 0.35***	[37]	7.0
	Distraction	Brief COPE		SF-36 PCS/SF-36 MCS		0.08^P^	−0.11^M^		[46]	4.0
	Active coping	Brief COPE		SF-36 PCS/SF-36 MCS		−0.16^P^	0.11^M^			
	Denial	Brief COPE		SF-36 PCS/SF-36 MCS		−0.15^P^	0.23^M^			
	Emotional support	Brief COPE		SF-36 PCS/SF-36 MCS		0.38^P^*	0.10^M^			
	Instrumental support	Brief COPE		SF-36 PCS/SF-36 MCS		−0.31^P^	−0.02^M^			
	Disengagement	Brief COPE		SF-36 PCS/SF-36 MCS		0.16^P^	0.33^M^			
	Venting	Brief COPE		SF-36 PCS/SF-36 MCS		−0.10^P^	−0.38*^M^			
	Positive reframing	Brief COPE		SF-36 PCS/SF-36 MCS		−0.22^P^	0.32^M^			
	Planning	Brief COPE		SF-36 PCS/SF-36 MCS		−0.23^P^	0.11^M^			
	Acceptance	Brief COPE		SF-36 PCS/SF-36 MCS		−0.18^P^	0.23^M^			
	Humor	Brief COPE		SF-36 PCS/SF-36 MCS		−0.15^P^	0.25^M^			
	Self-blame	Brief COPE		SF-36 PCS/SF-36 MCS		0.11^P^	−0.24^M^			
	Substance use	Brief COPE		SF-36 PCS/SF-36 MCS		0.26^P^	−0.44*^M^			
	Problem management	MNDCS^2^	Global QoL	SEIQoL index score				β = 0.44**	[36]	6.0
	Problem management	MNDCS^2^		SEIQoL index score	3-6 mo F.U.	nr		β = 0.42**	[30]	6.0
	Problem appraisal	MNDCS^2^		SEIQoL index score		nr		β = 0.15	[36]	6.0
	Emotion management	MNDCS^2^		SEIQoL index score		nr		β = −0.26		
	Emotional avoidance	MNDCS^2^		SEIQoL index score		nr		β = 0.39*		
	Emotional avoidance	MNDCS^2^		SEIQoL index score	3–6 mo F.U.	nr		β = 0.28	[30]	6.0
	Hopelessness	BHS	Global QoL	Single-item-question^3^		0.43***		s**/nr	[45]	5.5
	Perception of burden to others	ZBI - *revised*	Global QoL	Single-item-question^3^		0.45***		s*/nr	[45]	5.5
	Cognitive appraisal									
	Appraisal total	Appraisal scale	Global QoL	SEIQoL index score		nr		*R* ^*2*^ = 2 %	[36]	6.0
	Primary appraisal	Appraisal scale		SEIQoL index score		nr		β = −0.004		
	Appraisal of coping potential	Appraisal scale		SEIQoL index score		nr		β = 0.15		
PERSONALITY	Agreeableness	NEO-FFI	Global QoL	Single-item-question^4^		nr		β = 1.88*	[27]	4.0
	Agreeableness	NEO-FFI	HRQoL	HRCS		nr		β = 0.69*		
	Agreeableness x time	NEO-FFI	Global QoL	Single-item-question^4^	*over* 12 mo	nr		β = −0.28*		
	Agreeableness x time	NEO-FFI	HRQoL	HRCS	*over* 12 mo	nr		β = −0.09*		
	Neuroticism	NEO-FFI	Global QoL	Single-item-question^4^		nr		ns/nr		
	Extraversion	NEO-FFI		Single-item-question^4^		nr		ns/nr		
	Openness	NEO-FFI		Single-item-question^4^		nr		ns/nr		
	Conscientiousness	NEO-FFI		Single-item-question^4^		nr		ns/nr		

*Note.* Significance levels: *p < 0.1; **p < 0.01; ***p < 0.001

Abbreviations: *ns* not significant, *nr* not reported, *r* correlation, *β* standardized regression coefficient, *R* ^2^ explained variance of the determinant, *D* diagnosis, *A* obtained from the author, *F.U*. follow-up, *1* modified version 1, *2* modified version 2, *3* self-developed single-item-question by Ganzini, *4* self-developed single-item-question by Krampe, *5* SWBS developed by Reed [51], *6* SWBS developed by Ellison [52], *HRCS* health-related QoL composite score, *PCS* physical component summary (P), *MCS* mental component summary (M), *T0* data from baseline measurement, *O* overall (both < 1 month after diagnosis and > 1 month after diagnosis); Abbreviations of measurements: see Tables 2, 3 and 4

### Mood associated with QoL

Concerning mood, relationships between QoL and anxiety, depression and confusion-bewilderment were found.

#### Anxiety

Six studies assessed the relationship between anxiety and QoL [15, 19, 37, 38, 40, 41]; three studies reported HRQoL and three global QoL.

Two out of three studies, including one high quality study [37] which used a modified HADS, showed significant relationships with HRQoL; anxiety was strongly (−0.53) negatively associated with HRQoL. A similar contribution was obtained from multivariate analyses [19]. In contrast, one high quality study failed to find any relationship between anxiety and HRQoL [40].

Significant negative associations with global QoL were found in one out of three studies [41]. More specifically, low trait and state anxiety was associated with higher global QoL and was found in the diagnostic as well as the follow-up phase [41]. Two studies, of which one used a modified HADS, did not find any significant associations with global QoL [15, 19].

#### Depression

In total, fourteen studies assessed depression in relation to QoL [10, 13, 15, 19, 27, 28, 37, 38, 40–45]. Eight studies reported associations with HRQoL and eight studies with global QoL. Two studies reported both HRQoL and global QoL outcomes.

A significantly negative association with depression and HRQoL was reported in seven out of eight studies, including two of high quality, of which one used a modified HADS. Depression was moderately to strongly (−0.430; −0.60; −0.617) [10, 37, 42] correlated with HRQoL [10, 27, 42–44]. The contribution of depressive symptoms to HRQoL was also endorsed in regression analysis [19, 27, 37, 43, 44]. A single study of high quality failed, however, to find a significant association with depression and HRQoL in bivariate correlations [40]. Another study used four different HRQoL measures and found two out of four significant associations between depression and HRQoL in bivariate and multivariate analysis [44].

A significantly negative association with depression and Global QoL was reported in four out of eight studies, in both bivariate and multivariate analysis [13, 27, 28, 41]. Depression was moderately (−0.36) [28] negatively associated with global QoL. In four other studies, however, including 2 which used ALS-specific questionnaires, no significant correlations between depression and global QoL were demonstrated [15, 38, 42, 45].

A prospective long-term follow-up study reported the relationship between depression and HRQoL and global QoL during the first year after baseline measurement. Patients who were more depressed had lower HRQoL and global QoL scores at month 1 and during a 12-month follow-up. The results of a linear mixed model analysis showed no interaction effect between depression and time, indicating that more depressed patients did not differ from less depressed patients as far as the trajectories of Global QoL and HRQoL were concerned [27].

#### Confusion-bewilderment

One study examined the relation between ‘confusion—bewilderment’ and QoL. In regression analysis, this mood state made a significant positive contribution to HRQoL (β = 0.33) [19].

### Beliefs associated with QoL

With regard to beliefs, relationships between QoL and religiosity, spirituality, mindfulness, coping, hopelessness, perception of burden and cognitive appraisal were found.

#### Religiosity

Five studies assessed the relationship between religiosity and QoL [13, 14, 16, 31, 46]: three of these reported HRQoL, four global QoL and two studies both HRQoL and global QoL outcomes.

Two out of three studies did not find any significant relationships between religiosity and HRQoL [14, 46]. Regression analyses of a third study, which was of high quality, [31] revealed that a high level of religiosity made a significant positive contribution to HRQoL at 6 months’ follow-up, but not at the earlier assessment (3 months’ follow-up).

Three out of four studies [13, 14, 16] showed that a higher level of religiosity was significantly related to higher global QoL. Both ‘religiosity’ and ‘private religiosity’ (how religious patients perceived themselves to be and the amount of strength and comfort obtained from religious practices) developed a significant, moderate to strong association with global QoL over time (3–16 months’ and 6–16 months’ follow-up, respectively) [16]. Regression analysis confirmed this increasing relationship between ‘private religiosity’ and global QoL with time and showed an increase in explained variance of 16 % at 12 months' follow-up [16]. On the other hand, one high quality study did not find any significant associations with religiosity and global QoL at 3 or 6 months’ follow-up [31].

#### Spirituality

Two studies tested the correlation between spirituality and QoL [16, 47]. One used an HRQoL measure [47], whereas the other a global QoL measure [16].

The first study [47] split spiritual well-being along the dimensions of religious well-being (which refers to a relationship with God or what is understood as a spiritual being) and existential well-being (which involves a sense of purpose and meaning in life as a means of feeling connected to the world, separate from any specifically religious reference, beliefs and needs). Existential well-being was not associated with HRQoL. In contrast, religious well-being was strongly associated (−0.99) with higher HRQoL, independent of the clinical phase of ALS.

The second study [16] showed that spirituality (which refers to a search for the sacred or divine through any type of life experience) was strongly associated (0.54) with higher global QoL at long-term follow-up (12–16 months) but not at the earlier assessments.

### Mindfulness

One recent high quality study found a positive association between mindfulness ‘the process of actively making new distinctions about a situation and its environment, or its current context, rather than relying on previous categorizations from the past’ [48] and global QoL [29]. The results of a linear mixed model analysis showed that high mindfulness at baseline predicted significantly higher global QoL scores after four months [29].

#### Coping

Four studies, including three high quality studies, investigated the associations between coping and QoL [30, 36, 37, 46], in which two studies reported HRQoL and two studies global QoL measures.

One high quality study out of two showed that ‘adoption of positive coping strategies’ was moderately positively (0.46) associated with HRQoL [37]. The second study [46] related 14 coping strategies to HRQoL (36-item Short Form (SF36); Mental Component Summary score (MCS) and Physical Component Summary scores (PCS)). Of these 28 bivariate correlations, three were significantly associated: negative, moderate correlations were noted between MCS and substance use (−0.44) and between MCS and venting (an externalizing coping technique, the outward expression of emotions) (−0.38). PCS was positively, moderately associated with emotional support (0.38). The other 11 coping strategies (e.g. acceptance, denial, self-blame) were not associated with HRQoL [46].

Two high quality studies, analyzing the same cohort, showed that the coping strategy ‘problem management’ was positively associated with global QoL. The first study [36] with a cross-sectional design, using multivariate regression analysis, revealed positive associations between the coping strategy ‘problem management’ and ‘emotional avoidance’ and global QoL at baseline [30]. Analysis of a longitudinal follow-up [30] revealed that only the coping strategy ‘problem management’ was a significant predictor; patients who searched more frequently for information and support at baseline reported higher global QoL at 3 to 6 months’ follow-up [30].

#### Hopelessness

One study tested the association of hopelessness and global QoL [45]. It was shown that greater hopelessness was moderately correlated with lower global QoL (0.43). This relationship was still significant in a multivariate regression analysis with control variables.

#### Perception of burden to others

A single study examined the ‘perception of burden to others’. Having the belief of being a burden to others was moderately associated with lower global QoL (0.45). The association remained significant in the regression analyses [45].

#### Cognitive appraisal

A single study [36] assessed ‘cognitive appraisal’, which was split into patient’s primary (motivational relevance, motivational congruence) and secondary appraisal (problem focused and emotional focused coping potential) and related to global QoL. Results of the regression analysis showed no associations of cognitive appraisal with global QoL. The variance of the global QoL scores could not be significantly accounted for by any of the appraisals scales.

### Personality associated with QoL

#### Neuroticism, extraversion, openness, agreeableness and conscientiousness

One study [27] investigated the relationship between personality factors and QoL. In the regression analysis, it was shown that among the five personality factors (neuroticism, extraversion, openness, agreeableness and conscientiousness), only agreeableness had a strong positive association with both global QoL and HRQoL. Agreeableness refers to ‘a personality trait manifesting itself in individual behavioral characteristics that are perceived as kind, sympathetic, cooperative, warm and considerate’ [49]. There was also a significant interaction effect of agreeableness and time, meaning that agreeableness significantly influenced the course of global QoL and HRQoL; patients who scored higher on agreeableness had higher QoL ratings at baseline measurement but their decline in QoL was steeper compared to patients with lower scores in agreeableness [27].

## Discussion

The aim of the present review was to systematically collect and appraise evidence of the relationships between psychological factors (mood, beliefs, personality) and QoL in patients with ALS. This review showed that higher levels of anxiety and depression appeared to be related to a poorer HRQoL, whereas a higher level of religiosity seemed to be related to higher global QoL. Furthermore, associations might change during the disease course.

### Mood

Anxiety seemed to be negatively related to HRQoL, because higher levels of anxiety were consistently associated with a poorer HRQoL. In contrast, global QoL showed no associations with anxiety; the association could not, however, be refuted because of one poor quality study. Depression was negatively associated with HRQoL, suggesting that the presence of depressive symptoms is related to a poorer HRQoL. On the other hand, for global QoL, we could not support a relationship with depression, because of inconsistent results.

Mood appeared to be related to HRQoL but not to global QoL. This is in concurrence with cancer studies [9, 50], which also revealed that depression explained a large amount of variation in HRQoL, but not global QoL. Our results might in part be ascribed to conceptual overlap [51] between determinants and outcomes. For example, questions about feelings of anxiety and depression are often also included in a HRQoL measure (e.g. “Have you felt downhearted and blue?” SF-36; question 9f [52]), and so studying anxiety and depression as determinants of HRQoL may result in strong associations between determinants and outcomes. This contamination is less likely between mood and global QoL measures, because global QoL assesses such a wide spectrum of domains that contribute to overall QoL as judged by the patient.

Furthermore, it is important to be aware that there is recent evidence suggesting that depression questionnaires, specifically the Beck Depression Inventory (BDI) [53] and to a lesser degree the Hospital Anxiety and Depression Scale (HADS) [54], tend to overestimate depression in ALS, since these scores are highly influenced by the physical impairment of the patients [55, 56]. Consequently, the relationships that have been found between depression and HRQoL is questionable.

### Beliefs

There seemed to be no relationship between religiosity and HRQoL, because most studies showed weak and non-significant associations. However, religiosity appeared to be positively associated with global QoL in the majority of the studies, including one high quality study. Consequently, we support the assumption that a high level of religiosity made a significant positive contribution to better global QoL. However, these results might also point out to contamination in concepts between religiosity and global QoL since the The McGill Quality of Life Questionnaire (MQOL) total score includes items about existential well-being.

Religiosity might be important for the individual’s global QoL because it may create meaning and coherence when an individual’s world is devastated by a distressing and progressive disease [47]. These findings are mirrored in other diseases such as Multiple Sclerosis [57] and advanced cancer [58]. It should be taken into account that most of the included studies about religiosity are from North America and the religiosity questionnaire which was used relies predominantly on monotheistic terminology, about belief in God or experience of God [59]. In current (western) culture, people are more interested in spirituality [60] and mindfulness [55] and are searching for a connection with the divine within themselves, instead of a connection with an external almighty power [60]. The fact that religiosity and spirituality are culture dependent and are defined differently in each country might explain the heterogeneity in findings on association of QoL.

### Personality

The search has yielded only one hit concerning personality factors, suggesting that personality factors were not considered to be the most important psychological factors influencing QoL. Only a single study of low quality was included, conclusive associations between HRQoL or global QoL could, therefore, not be established.

### Miscellaneous

Several other psychological factors were reported in only a single study or measured twice in the same cohort [30, 36]: there was, therefore, insufficient evidence to support associations between HRQoL or global QoL and the following psychological factors: confusion-bewilderment (mood); spirituality, mindfulness, coping styles, hopelessness, perception of burden, cognitive appraisal (beliefs) and neuroticism, extraversion, openness, agreeableness and conscientiousness (personality).

### Associations throughout the disease course

Although there was not enough evidence per psychological factor, it is valuable to point out with regard to psychological factors as a whole, that associations might change throughout the course of the disease; religiosity and spirituality appeared to become more positively associated with global QoL over time. This is in accordance with the theory of Waldron [61] who suggested that psychological adaptation to terminal illness may involve a shift in focus of determinants of QoL; in the initial stages of a progressive illness, patients may focus on physical functioning and on decreasing disability, but as the illness progresses, the importance of these issues may be replaced by a focus on the psychosocial and spiritual domains.

### Strengths and limitations of this systematic review

This is the first systematic review on associations between psychological factors and QoL in patients with ALS. This review was carried out in accordance with the PRISMA guidelines (see online Additional file 1). The methodological quality of the studies and the consistency of the associations between psychological factors and HRQoL and global QoL were comprehensively appraised. A limitation, however, of our study is that only a small number of psychological factors could be compared, because most of the associations with QoL were only reported once. Besides, our review may have missed relevant papers published in non-English journals. Finally, as studies with significant results are more likely to be published than studies without significant results, publication bias has to be taken into account.

### Limitations of the literature

First, the heterogeneity of the literature with respect to instruments for assessing psychological factors and QoL may have influenced the associations. Levels of anxiety and depression, for example, were measured using 5 and 7 different questionnaires, respectively, and moreover, with a mix of both generic and ALS-specific questionnaires, or questionnaires modified for ALS. Concerning QoL measures, 7 different HRQoL and 5 different global QoL (mostly generic) measures, were extracted. Second, in order to detect which psychological factors affect HRQoL and global QoL over the course of the disease, it is essential to cluster the data of patients according to the same disease stage (diagnostic stage, rehabilitation stage and terminal stage [62]). In fact, only one study analyzed the determinants of patients in the diagnostic phase separately [41]. Other longitudinal studies only reported changing associations of depression, coping, religiosity and personality factors after baseline measurement, without specific information about the disease stage. Other limitations concern studies with a cross-sectional design and the overrepresentation of small studies, without an adequate sample size in relation to the number of determinants.

## Conclusions, clinical implications and further research

Our results suggest that higher levels of anxiety and depression are related to a poorer HRQoL, whereas higher levels of religiosity appeared to be related to better global QoL. Furthermore, associations might change throughout the disease course.

Therefore it is important for health professionals to become aware of the relationships between psychological factors and QoL, as these relationships identify possible targets for interventions to improve QoL. It seems relevant for health professionals in ALS care, to focus on influencing mood and beliefs in order to improve HRQOL and global QoL. Furthermore, it is relevant to make a distinction between HRQoL and global QoL, because HRQoL is expected to decline, according to a decrease in mental and physical functioning, whereas global QoL seems more dependent on other factors, such as existential concerns.

More high quality research is needed to confirm the assumed association between anxiety, depression and religiosity and HRQoL and global QoL and to investigate how and when these factors can be targeted in ALS care. Coping, spirituality, mindfulness, hopelessness, perception of burden and agreeableness might be other promising factors that influence QoL, but warranted further investigation. More longitudinal studies in larger samples are needed because they allow causal relationships and effects of time to be identified. Furthermore, uniformity of measures for QoL and psychological factors, preferably ALS-specific, are required in order to obtain reliable, comparable data. As small sample sizes are inherent to ALS research, the answer may lie in international collaboration and data gathered by online survey.

## Additional files

Additional file 1: Prisma Checklist (DOC 64 kb) Additional file 2: PubMed Search (DOC 34 kb)
