# Supplementary material for: Associations between psychological factors and health-related quality of life and global quality of life in patients with ALS: a systematic review
Source: Health Qual Life Outcomes. 2016 Jul 20;14:107. doi: 10.1186/s12955-016-0507-6 (PMC4955215; doi:10.1186/s12955-016-0507-6)
Supplement: Additional file 2: — PubMed Search (DOC 34 kb) [file 12955_2016_507_MOESM2_ESM.doc]

**Additional file 2. Literature search**

**PubMed search strategy d.d. 13.10.2015**

**ALS domain 25.687**

"als"[Title/Abstract] OR "amyotrophic lateral sclerosis"[Title/Abstract] OR "mnd"[Title/Abstract] OR "motor neuron disease"[Title/Abstract] OR "motor neuron diseases"[Title/Abstract] OR Lou Gehrig's disease[Title/Abstract] OR als amyotrophic lateral sclerosis[MeSH Terms] OR amyotrophic lateral sclerosis[MeSH Terms]

**Psychological factor determinant 1.923.630**

"psychologic factor"[Title/Abstract] OR "psychologic factors"[Title/Abstract] OR "psychological factor"[Title/Abstract] OR "psychological factors"[Title/Abstract] OR "psychologic variable"[Title/Abstract] OR "psychologic variables"[Title/Abstract] OR "psychological variable"[Title/Abstract] OR "psychological variables"[Title/Abstract] OR "personal characteristic"[Title/Abstract] OR "personal characteristics"[Title/Abstract] OR "individual characteristics"[Title/Abstract] OR "personality traits"[Title/Abstract] OR "personality"[Title/Abstract] OR "psychosocial factor"[Title/Abstract] OR "psychosocial factors"[Title/Abstract] OR "psychologic function"[Title/Abstract] OR "psychologic functioning"[Title/Abstract] OR "psychological function"[Title/Abstract] OR "psychological functioning"[Title/Abstract] OR "individuality"[Title/Abstract] OR "coping"[Title/Abstract] OR "coping skill"[Title/Abstract] OR "coping skills"[Title/Abstract] OR "coping behaviour"[Title/Abstract] OR "coping behaviours"[Title/Abstract] OR "coping style"[Title/Abstract] OR "coping styles"[Title/Abstract] OR "psychological adjustment"[Title/Abstract] OR "psychological adjustments"[Title/Abstract] OR "psychologic adaptation"[Title/Abstract] OR "adaptive behaviour"[Title/Abstract] OR "adaptive behaviours"[Title/Abstract] OR "self assessment"[Title/Abstract] OR "appraisal"[Title/Abstract] OR "appraisals"[Title/Abstract] OR "mental state"[Title/Abstract] OR "mental status"[Title/Abstract] OR "disease attributes"[Title/Abstract] OR "body image"[Title/Abstract] OR "locus of control"[Title/Abstract] OR "internal external control"[Title/Abstract] OR "resilience"[Title/Abstract] OR "emotional stability"[Title/Abstract] OR "self blame"[Title/Abstract] OR "self efficacy"[Title/Abstract] OR "self esteem"[Title/Abstract] OR "self concept"[Title/Abstract] OR "self perception"[Title/Abstract] OR "mastery"[Title/Abstract] OR "optimism"[Title/Abstract] OR "pessimism"[Title/Abstract] OR "hope"[Title/Abstract] OR "positive affect"[Title/Abstract] OR "negative affect"[Title/Abstract] OR "negativism"[Title/Abstract] OR "affect"[Title/Abstract] OR "sense of coherence"[Title/Abstract] OR "purpose in life"[Title/Abstract] OR "personal autonomy"[Title/Abstract] OR "personal growth"[Title/Abstract] OR "five factor model"[Title/Abstract] OR "big five"[Title/Abstract] OR "big 5"[Title/Abstract] OR "openness"[Title/Abstract] OR "conscientiousness"[Title/Abstract] OR "introversion"[Title/Abstract] OR "extraversion"[Title/Abstract] OR "neuroticism"[Title/Abstract] OR "agreeableness"[Title/Abstract] OR "illness cognition"[Title/Abstract] OR "illness cognitions"[Title/Abstract] OR "acceptance"[Title/Abstract] OR "assertiveness"[Title/Abstract] OR "empathy"[Title/Abstract] OR "emotions"[Title/Abstract] OR "anger"[Title/Abstract] OR "anxiety"[Title/Abstract] OR "depression"[Title/Abstract] OR "fear"[Title/Abstract] OR "mood"[Title/Abstract] OR "grief"[Title/Abstract] OR "loneliness"[Title/Abstract] OR "panic"[Title/Abstract] OR "irritability"[Title/Abstract] OR "rage"[Title/Abstract] OR "catastrophizing"[Title/Abstract] OR "apathy"[Title/Abstract] OR "bereavement"[Title/Abstract] OR "boredom"[Title/Abstract] OR "euphoria"[Title/Abstract] OR "frustration"[Title/Abstract] OR "guilt"[Title/Abstract] OR "shame"[Title/Abstract] OR "happiness"[Title/Abstract] OR "hate"[Title/Abstract] OR "jealousy"[Title/Abstract] OR "laughter"[Title/Abstract] OR "love"[Title/Abstract] OR "pleasure"[Title/Abstract] OR "attitude"[Title/Abstract] OR "attitudes"[Title/Abstract] OR "beliefs"[Title/Abstract] OR "expectation"[Title/Abstract] OR "expectations"[Title/Abstract] OR "hopeless"[Title/Abstract] OR "illness perception"[Title/Abstract] OR "illness perceptions"[Title/Abstract] OR "motivation"[Title/Abstract] OR "motivations"[Title/Abstract] OR "representation"[Title/Abstract] OR "representations"[Title/Abstract] OR "religiosity"[Title/Abstract] OR "spirituality"[Title/Abstract] OR "thoughts"[Title/Abstract] OR "stress"[Title/Abstract] OR "awareness"[Title/Abstract] OR "imagination"[Title/Abstract] OR "intuition"[Title/Abstract]

**Quality of life outcome 374.750**

"quality of life"[Title/Abstract] OR "qol"[Title/Abstract] OR "life quality"[Title/Abstract] OR "life qualities"[Title/Abstract] OR "hrqol"[Title/Abstract] OR "hql"[Title/Abstract] OR "health related quality of life"[Title/Abstract] OR "well being"[Title/Abstract] OR "value of life"[Title/Abstract] OR "livability"[Title/Abstract] OR "perceived health"[Title/Abstract] OR "sanctity of life"[Title/Abstract] OR "health status"[Title/Abstract] OR "well being"[Title/Abstract] OR "wellbeing"[Title/Abstract] OR "quality of life"[MeSH Terms] OR health status[MeSH Terms]

**Domain AND Determinant AND Outcome = 280**

**Search** (((("quality of life"[Title/Abstract] OR "qol"[Title/Abstract] OR "life quality"[Title/Abstract] OR "life qualities"[Title/Abstract] OR "hrqol"[Title/Abstract] OR "hql"[Title/Abstract] OR "health related quality of life"[Title/Abstract] OR "well being"[Title/Abstract] OR "value of life"[Title/Abstract] OR "livability"[Title/Abstract] OR "perceived health"[Title/Abstract] OR "sanctity of life"[Title/Abstract] OR "health status"[Title/Abstract] OR "well being"[Title/Abstract] OR "wellbeing"[Title/Abstract] OR "quality of life"[MeSH Terms] OR health status[MeSH Terms]))) AND (("psychologic factor"[Title/Abstract] OR "psychologic factors"[Title/Abstract] OR "psychological factor"[Title/Abstract] OR "psychological factors"[Title/Abstract] OR "psychologic variable"[Title/Abstract] OR "psychologic variables"[Title/Abstract] OR "psychological variable"[Title/Abstract] OR "psychological variables"[Title/Abstract] OR "personal characteristic"[Title/Abstract] OR "personal characteristics"[Title/Abstract] OR "individual characteristics"[Title/Abstract] OR "personality traits"[Title/Abstract] OR "personality"[Title/Abstract] OR "psychosocial factor"[Title/Abstract] OR "psychosocial factors"[Title/Abstract] OR "psychologic function"[Title/Abstract] OR "psychologic functioning"[Title/Abstract] OR "psychological function"[Title/Abstract] OR "psychological functioning"[Title/Abstract] OR "individuality"[Title/Abstract] OR "coping"[Title/Abstract] OR "coping skill"[Title/Abstract] OR "coping skills"[Title/Abstract] OR "coping behaviour"[Title/Abstract] OR "coping behaviours"[Title/Abstract] OR "coping style"[Title/Abstract] OR "coping styles"[Title/Abstract] OR "psychological adjustment"[Title/Abstract] OR "psychological adjustments"[Title/Abstract] OR "psychologic adaptation"[Title/Abstract] OR "adaptive behaviour"[Title/Abstract] OR "adaptive behaviours"[Title/Abstract] OR "self assessment"[Title/Abstract] OR "appraisal"[Title/Abstract] OR "appraisals"[Title/Abstract] OR "mental state"[Title/Abstract] OR "mental status"[Title/Abstract] OR "disease attributes"[Title/Abstract] OR "body image"[Title/Abstract] OR "locus of control"[Title/Abstract] OR "internal external control"[Title/Abstract] OR "resilience"[Title/Abstract] OR "emotional stability"[Title/Abstract] OR "self blame"[Title/Abstract] OR "self efficacy"[Title/Abstract] OR "self esteem"[Title/Abstract] OR "self concept"[Title/Abstract] OR "self perception"[Title/Abstract] OR "mastery"[Title/Abstract] OR "optimism"[Title/Abstract] OR "pessimism"[Title/Abstract] OR "hope"[Title/Abstract] OR "positive affect"[Title/Abstract] OR "negative affect"[Title/Abstract] OR "negativism"[Title/Abstract] OR "affect"[Title/Abstract] OR "sense of coherence"[Title/Abstract] OR "purpose in life"[Title/Abstract] OR "personal autonomy"[Title/Abstract] OR "personal growth"[Title/Abstract] OR "five factor model"[Title/Abstract] OR "big five"[Title/Abstract] OR "big 5"[Title/Abstract] OR "openness"[Title/Abstract] OR "conscientiousness"[Title/Abstract] OR "introversion"[Title/Abstract] OR "extraversion"[Title/Abstract] OR "neuroticism"[Title/Abstract] OR "agreeableness"[Title/Abstract] OR "illness cognition"[Title/Abstract] OR "illness cognitions"[Title/Abstract] OR "acceptance"[Title/Abstract] OR "assertiveness"[Title/Abstract] OR "empathy"[Title/Abstract] OR "emotions"[Title/Abstract] OR "anger"[Title/Abstract] OR "anxiety"[Title/Abstract] OR "depression"[Title/Abstract] OR "fear"[Title/Abstract] OR "mood"[Title/Abstract] OR "grief"[Title/Abstract] OR "loneliness"[Title/Abstract] OR "panic"[Title/Abstract] OR "irritability"[Title/Abstract] OR "rage"[Title/Abstract] OR "catastrophizing"[Title/Abstract] OR "apathy"[Title/Abstract] OR "bereavement"[Title/Abstract] OR "boredom"[Title/Abstract] OR "euphoria"[Title/Abstract] OR "frustration"[Title/Abstract] OR "guilt"[Title/Abstract] OR "shame"[Title/Abstract] OR "happiness"[Title/Abstract] OR "hate"[Title/Abstract] OR "jealousy"[Title/Abstract] OR "laughter"[Title/Abstract] OR "love"[Title/Abstract] OR "pleasure"[Title/Abstract] OR "attitude"[Title/Abstract] OR "attitudes"[Title/Abstract] OR "beliefs"[Title/Abstract] OR "expectation"[Title/Abstract] OR "expectations"[Title/Abstract] OR "hopeless"[Title/Abstract] OR "illness perception"[Title/Abstract] OR "illness perceptions"[Title/Abstract] OR "motivation"[Title/Abstract] OR "motivations"[Title/Abstract] OR "representation"[Title/Abstract] OR "representations"[Title/Abstract] OR "religiosity"[Title/Abstract] OR "spirituality"[Title/Abstract] OR "thoughts"[Title/Abstract] OR "stress"[Title/Abstract] OR "awareness"[Title/Abstract] OR "imagination"[Title/Abstract] OR "intuition"[Title/Abstract]))) AND (("als"[Title/Abstract] OR "amyotrophic lateral sclerosis"[Title/Abstract] OR "mnd"[Title/Abstract] OR "motor neuron disease"[Title/Abstract] OR "motor neuron diseases"[Title/Abstract] OR Lou Gehrig's disease[Title/Abstract] OR als amyotrophic lateral sclerosis[MeSH Terms] OR amyotrophic lateral sclerosis[MeSH Terms]))

**= 280 articles**
